# Antibody-Drug Conjugates Containing Payloads from Marine Origin

**DOI:** 10.3390/md20080494

**Published:** 2022-07-30

**Authors:** Iván Cheng-Sánchez, Federico Moya-Utrera, Cristina Porras-Alcalá, Juan M. López-Romero, Francisco Sarabia

**Affiliations:** 1 Department of Chemistry, University of Zurich, Winterthurerstrasse 190, 8057 Zurich, Switzerland; 2 Department of Organic Chemistry, Faculty of Sciences, University of Málaga, 29071 Málaga, Spain; fedemu@uma.es (F.M.-U.); cristinaalcala@uma.es (C.P.-A.); jmromero@uma.es (J.M.L.-R.); frsarabia@uma.es (F.S.)

**Keywords:** antibody-drug conjugates, anticancer compounds, marine natural product, bioactive compounds

## Abstract

Antibody-drug conjugates (ADCs) are an important class of therapeutics for the treatment of cancer. Structurally, an ADC comprises an antibody, which serves as the delivery system, a payload drug that is a potent cytotoxin that kills cancer cells, and a chemical linker that connects the payload with the antibody. Unlike conventional chemotherapy methods, an ADC couples the selective targeting and pharmacokinetic characteristics related to the antibody with the potent cytotoxicity of the payload. This results in high specificity and potency by reducing off-target toxicities in patients by limiting the exposure of healthy tissues to the cytotoxic drug. As a consequence of these outstanding features, significant research efforts have been devoted to the design, synthesis, and development of ADCs, and several ADCs have been approved for clinical use. The ADC field not only relies upon biology and biochemistry (antibody) but also upon organic chemistry (linker and payload). In the latter, total synthesis of natural and designed cytotoxic compounds, together with the development of novel synthetic strategies, have been key aspects of the consecution of clinical ADCs. In the case of payloads from marine origin, impressive structural architectures and biological properties are observed, thus making them prime targets for chemical synthesis and the development of ADCs. In this review, we explore the molecular and biological diversity of ADCs, with particular emphasis on those containing marine cytotoxic drugs as the payload.

## 1. Introduction

**Figure 1:**
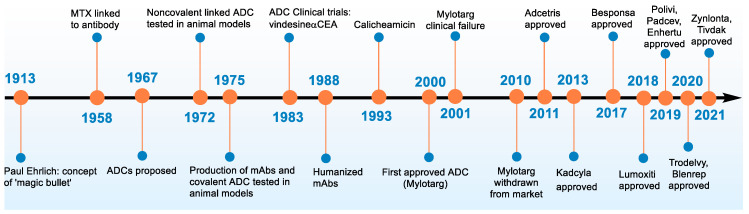
ADC timeline: from the concept to the clinic.

Antibody-drug conjugates (ADCs) are targeted therapeutics that have been developed for cancer treatment by delivering a potent cytotoxic drug selectively to cancer cells. The concept of ADCs was first introduced more than a century ago by the German Nobel laureate Paul Ehrlich who had the idea of a ‘magic bullet’ as a chemotherapy [1]. Ehrlich’s idea relied on killing specific targets that cause diseases without affecting the body itself. In other words, he postulated a strategy to efficiently target cancer cells with high precision and specificity. His research to discover the ‘magic bullet’ resulted in further knowledge of the functions of the body’s immune system. With the major advances in immunology and organic synthesis, it took decades for Ehrlich’s idea to become a reality, represented today by the ADCs depicted [2] (Figure 1).

Intense research efforts have been devoted to the design, synthesis, and development of ADCs with the aim of market ADC-based drugs for cancer treatment. First generations of ADCs used clinically approved drugs, such as methotrexate (MTX), 5-fluorouracil, mitomycin, or vinblastine [3]; however, the low drug potency, high antigen expression on normal cells, and the low stability of the linker resulted in failure for human clinical use [4]. By further exploring targets, antibodies, linker optimization, and conjugation strategies, finally, in 2000, the first ADC to be approved by the U.S. Food and Drug Administration (FDA) was Mylotarg^®^ (Pfizer, gemtuzumab ozogamicin) [5,6]. However, it was withdrawn in the USA in 2010 for efficacy and overall survival problems and later reapproved in 2017 under a new regimen used against acute myeloid leukemia. In 2011, Adcetris^®^ (Seattle Genetics, Brentuximab Vedotin), the second FDA-approved ADC, came to the market and was used against relapsed or refractory Hodgkin lymphoma and systemic anaplastic large-cell lymphoma [7,8]. The next ADC launched was Kadcyla^®^ (Roche, Trastuzumab Emtansine) in 2013 for the treatment of HER2-positive breast cancers [9,10]. In 2017, the FDA approved Besponsa^®^ (Pfizer, inotuzumab ozogamicin) used against relapsed and refractory B-cell precursor acute lymphoblastic leukemia [11]. After these milestones in cancer therapy, eight more ADCs have been approved by FDA in the last few years: Lumoxiti^®^ (2018, Astrazeneca, moxetumomab pasudotox) [12], Polivy^®^ (2019, Genentech, Roche, polatuzumab vedotin-piiq) [13], Padcev^®^ (2019, Astellas/Seattle Genetics, enfortumab vedotin) [14], Enhertu^®^ (2019, AstraZeneca/Daiichi Sankyo, Trastuzumab deruxtecan) [15], Trodelvy^®^ (2020, Immunomedics, sacituzumab govitecan) [16], Blenrep^®^ (2020, GlaxoSmithKline, belantamab mafodotin-blmf) [17], Zynlonta (2021, ADC Therapeutics, loncastuximab tesirine-lpyl) [18] and Tivdak (2021, Seagen Inc, tisotumab vedotin-tftv) [19]. Currently, over 100 ADCs are in clinical trials, highlighting the importance of ADCs in the development of targeted cancer therapies [20] (Figure 1). 

**Figure 2:**
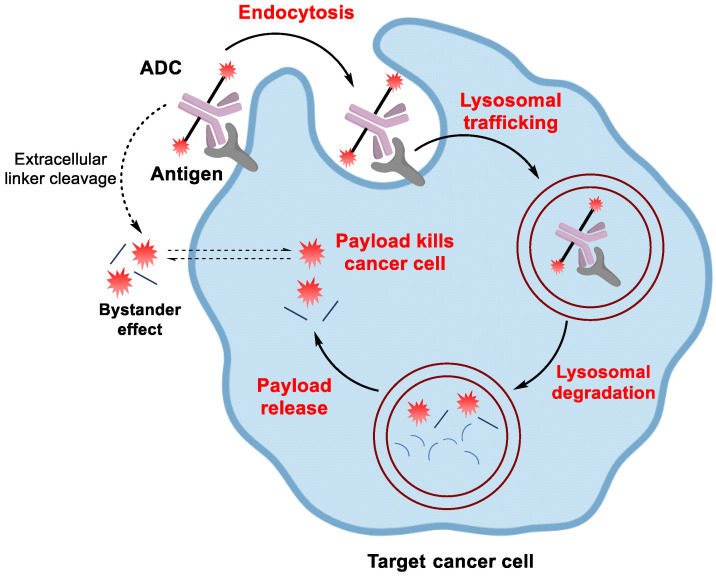
Antibody-drug conjugate mechanism of action.

ADCs combine the advantages of both antibody and the payload (the cytotoxic drug), coupling the targeting and pharmacokinetic characteristics associated with the antibody with the high capacity of killing cancer cells by the payload. In contrast to conventional chemotherapy methods, which lack target selectivity and damage healthy cells, ADCs deliver the potent anticancer drug via a chemical linker conjugated to a monoclonal antibody (mAb), which is associated with a specific cancer cell type. The antibody of the ADC binds to an antigen that is expressed at higher levels in cancer cells compared to healthy tissue [21]. Once bound, the ADC-antigen complex is internalized into the cancer cell via endocytosis, and then, after lysosome trafficking and degradation, it can be dismantled, releasing the payload, thus selectively destroying the target cancer cell and minimizing off-target effects [22]. If the payload has enough membrane permeability, it can diffuse to adjacent cancer cells in a tumor to kill surrounding cells (‘bystander effect’) that may or may not express the ADC target antigen [23]. Unlike traditional drugs, this mechanism of action has the advantage of high specificity and potency, reducing off-target toxicities in patients by limiting the exposure of healthy tissues to the cytotoxic drug (Figure 2). However, it is important to point out that the selectivity of ADCs on cancer cells is limited by several factors that are responsible for their toxicity: (1) linker-drug instability that contributes to the premature release of payload in circulation (toxicity is payload dependent—‘off-target, off-tumor’); (2) if the target antigen is expressed in non-malignant cells this affects the distribution of the cytotoxic drug and where it accumulates, leading to toxicity that is not payload dependent—‘on-target, off-tumor’; and (3) uptake of ADCs into non-malignant cells by binding to Fc receptors (FcγRs, FcRn, and C-type lectin receptors), and thorough nonspecific endocytosis (macro- and micropinocytosis) mechanisms [24,25].

### 1.1. ADC Design

**Figure 3:**
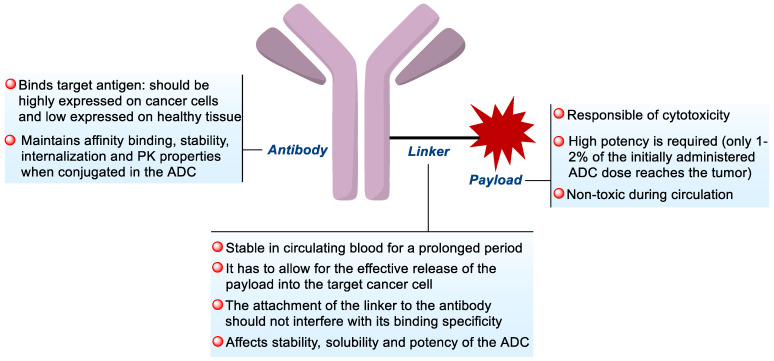
General structure of antibody-drug conjugate.

The structure of an ADC comprises three main domains: the monoclonal antibody, the linker, and the payload (Figure 3). These components are key for the pharmacology of the resulting ADC, and careful selection and optimization of the different domains are required for success. 

#### 1.1.1. Antibody

The antibody recognizes and binds to the antigens on the target cancer cell, carrying the linked cytotoxic payload to the tumor site. To maximize the potency of the ADC while limiting off-target toxicity, the choice of the antibody should be based on a well-characterized antigen, ideally abundantly expressed at the tumor sites and low or no expression in normal tissues [26]. However, antigen expression in normal tissues can be tolerated if its expression on vital organs is minimal or absent [27]. 

Another important factor is the efficiency of the internalization of the ADC-antigen complex, which relies on the affinity of the binding of the antibody with the antigen. Sufficient affinity is required for rapid internalization of the ADC-antigen complex; however, antibodies with high antigen affinity may compromise the penetration into solid tumors [20]. The most commonly used monoclonal antibodies in ADCs are human IgG isotypes and, in particular, IgG1, as it possesses a long half-life and is able to generate stronger antibody-dependent cell-mediated cytotoxicity and complement-dependent toxicity toward cancer cells [28,29].

#### 1.1.2. Linker

The linker plays an essential role in releasing the potent cytotoxic drug into the cancer cells [30]. To prepare selective and potent ADCs, the linker should possess the following characteristics: (1) it needs to be stable enough in circulating blood for a prolonged period to avoid the premature release of the cytotoxic drug and avoid off-target effects, but at the same time it has to allow for the effective release of the payload into the target cancer cell; (2) the linker has to ensure that the ADC is soluble, therefore high water solubility is required for bioconjugation; (3) the antibody must retain its function; thus, the attachment of the linker to the antibody should not interfere with its binding specificity.

There are two different parts to the linkers: the antibody- and the payload-attachment sites. The site of conjugation and choice of the linker is critical for the stability and pharmacokinetic properties of the ADCs. The stoichiometry of the linker-payloads on the antibody (drug-to-antibody ratio, DAR) determines the homogeneity and stability of the ADC [31]. The bonding between the linker and the payload is also critical and has been proven to be a key requirement for safe and efficacious ADCs [32].

The linkers can be classified into two groups based on the release mechanism and their stability in circulation: (1) cleavable and (2) noncleavable. Cleavable linkers release the drug to the target cell using the physiological environment [33] and can be divided into three main groups: (1) acid-cleavable linkers, such as a hydrazone, that release the drug at the low pH of the lysosome; (2) reducible linkers, that contain a disulfide bond that will be reduced by glutathione to release the drug, taking advantage of the higher intracellular glutathione concentration in cancer cells; and (3) enzyme cleavable linkers, that contain sensitive functional groups that will be recognized by proteases, phosphatases, glycosidases or sulfatases, and therefore allow the release of the drug into the lysosome. On the other hand, noncleavable linkers are based on the degradation of the antibody into the corresponding amino acids during metabolism in the lysosome, releasing an amino acid carrying the linker and the drug [30]. Noncleavable linkers are more stable in circulating blood than cleavable linkers, but they are not able to kill neighboring cancer cells by bystander effect. This is explained by the lack of cell permeability related to the charged amino acid appendage. In contrast, cleavable linkers enhance the bystander effect and can distinguish between the circulatory and target cells conditions, making them the preferred choice to treat the majority of cancer types [33].

#### 1.1.3. Payload

Payloads used in ADCs have to possess several requirements. Ideally, it should be a very potent cytotoxic drug with potency in the sub-nanomolar range. This requirement is due to the low intracellular concentration of the cytotoxic payload in cancer cells. Indeed, only 1–2% of the initially administered ADC dose reaches the tumor, mainly because of limitations such as distribution into tumor tissues or efficient internalization and delivery [34]. However, potency is not the only requirement, and the molecular architecture of the payload is also very important. The payload molecule should contain functional groups compatible with the attachment of a chemical linker, which connects to the antibody. Solubility of the cytotoxic molecule also has to be considered since the payload should be soluble enough to allow conjugation to the antibody in aqueous buffers [35]. However, lipophilic payloads are necessary to pass cell membranes and later escape from the lysosome in the release. A balance between these factors can be achieved by modification of the functional groups in the linker. Another important factor is the payload stability during circulation, uptake, and release to the target. For example, acid-sensitive payloads are unstable in the lysosome, while payloads containing functional groups such as a disulfide, alkene, or epoxides may be reduced or transformed by enzymes, preventing the delivery of the cytotoxic drug to the final target [36].

**Figure 4:**
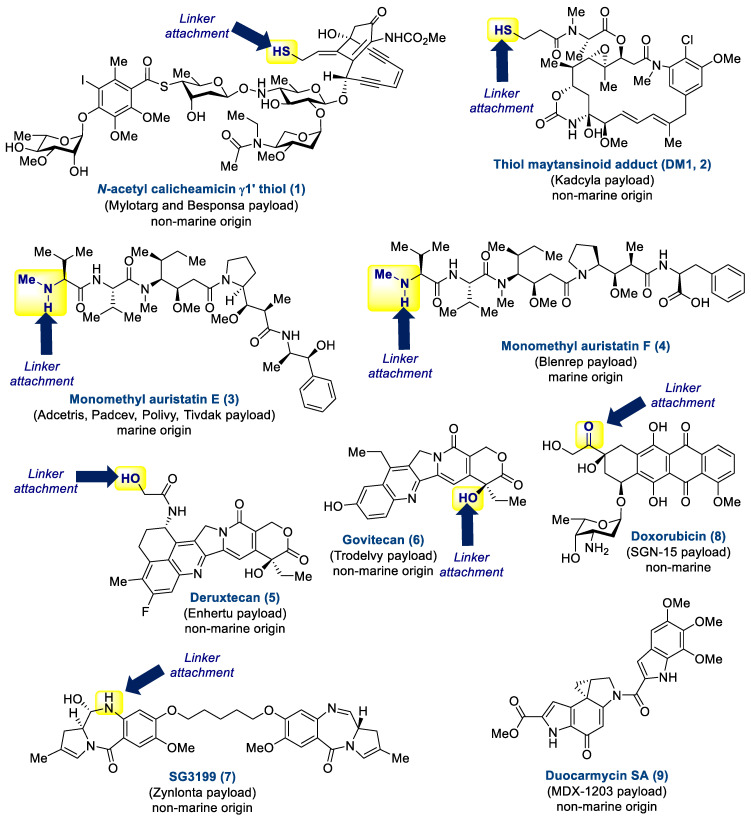
Selected payloads used in antibody-drug conjugates from marine and non-marine origin.

There are two main groups of cytotoxic drugs employed in the ADC field. The first class contains microtubule inhibitors that disrupt microtubule assembly and affect mitosis [37]. Typical payloads belonging to this group are dolastatin 10-based auristatin analogs **3** and **4** (Adcetris, Padcev, Polivy, Tivdak, and Blenrep) and maytansinoids such as DM1 (**2**) (Kadcyla) (Figure 4 and Table 1). For those tumors that are not sensitive to tubulin-disrupting agents, a second group of payloads has been developed, consisting of DNA-damaging drugs [38]. Duocarmycin analogs, such as **9** and its derivatives (MDX-1203 and SYD-985), cause apoptotic cell death by selective alkylation of adenine-N3 in the minor groove of DNA [39]. Calicheamicin derivatives such as **1**, a potent antitumor antibiotic, also bind the minor groove of DNA, causing double-strand DNA breaks and cell death [40] (Figure 4). Other payloads under investigation are α-amanitin (RNA polymerase II inhibitor) [41], irinotecan (topoisomerase inhibitor) [42], and pyrrolobenzodiazepines (which bind to discrete DNA sequences causing lethal lesions) [43], of which payloads **5**–**9** are representative examples employed in ADCs (Figure 4 and Table 1).

Given the biological relevance and structural complexity of these classes of cancer therapeutics, many reviews have been devoted to all aspects of the ADCs, including general details [20,21,24,25,26,28,29,34,35,36,37,38,44,45,46,47,48,49,50,51,52,53,54,55], the role of the linker [30,32,33] as well as the clinical status of ADCs [56,57,58,59]. As described in the Introduction, ADCs rely on several fields: the antibody belongs to biology and biochemistry, while the linker and payload fall into organic chemistry. Although the latter is very important for ADC design, only a few reviews have been published focusing on the role of organic synthesis in the ADC field [60,61]. More specifically, in the case of ADCs containing payloads from marine origin, a review was published in 2017 [62], which provided a general description of ADCs, with emphasis on natural toxins, especially from marine origin, but lacking synthetic chemistry details.

Considering the publication landscape, the current review intends to provide a chemical and biological perspective of these fascinating cancer therapeutics with a particular emphasis on ADCs containing payloads of marine origin that have not been covered in the aforementioned reviews. This review also provides an update on the state of the art in this field, which has attracted the interest of chemists and biologists, revealing the potential that these compounds can provide in biology, chemistry, and biomedicine.

## 2. Chemistry and Biology of Marine Antibody-Drug Conjugates

### 2.1. Antibody-Drug Conjugates Based on the Auristatins

The discovery of dolastatin 10 (**10**), isolated from the sea hare *Dolabella auricularia* by Pettit et al. in 1987 [63], followed by its structural determination and recognition of its striking antitumor properties [64] ushered in a fascinating new chapter about the value and significance of natural products in the development of new anticancer compounds [65]. Thus, the disclosure of its impressive inhibitory activities in the pM range against a variety of NCI human cancer cell lines was followed by the determination of its mechanism of action, featured by its ability to inhibit tubulin polymerization, binding at the vinca alkaloid sites in a noncompetitive manner [66]. An extensive structure-activity relationship study, through modifications of its different structural units, consisting of dolavaline (Dov), valine (Val), dolaleuine (Dil), dolaproine (Dap), and dolaphenine (Doe), allowed for the determination of the structural requirements to maintain their cytotoxic activities [67]. In addition, the SAR study led to improved solubility and reduced toxicity, which represented the main pharmacologic hurdle that has hampered its clinical development and approval as a cancer therapy [68]. Among all the described analogs, auristatin E (**11**), in which the Doe unit is replaced with (1*S*, 2*R*)-2-amino-1-phenylpropan-1-ol, was identified as one of the best analogs of dolastatin 10 (**10**) [69]. In addition, the identification of the monomethyl amino dolastatin 10 (MMAD, **12**) as a potent analog as active as dolastatin 10 (**10**) opened the opportunity to use this site for the linkage of specific antibodies capable of recognizing tumor antigens [70,71]. Thus, encouraged by the approval of Mylotarg in 2000 for the treatment of acute myeloid leukemia, SeaGen initiated a research program directed toward the design and synthesis of ADCs based on auristatin E (**11**) [8]. 

**Scheme 1:**
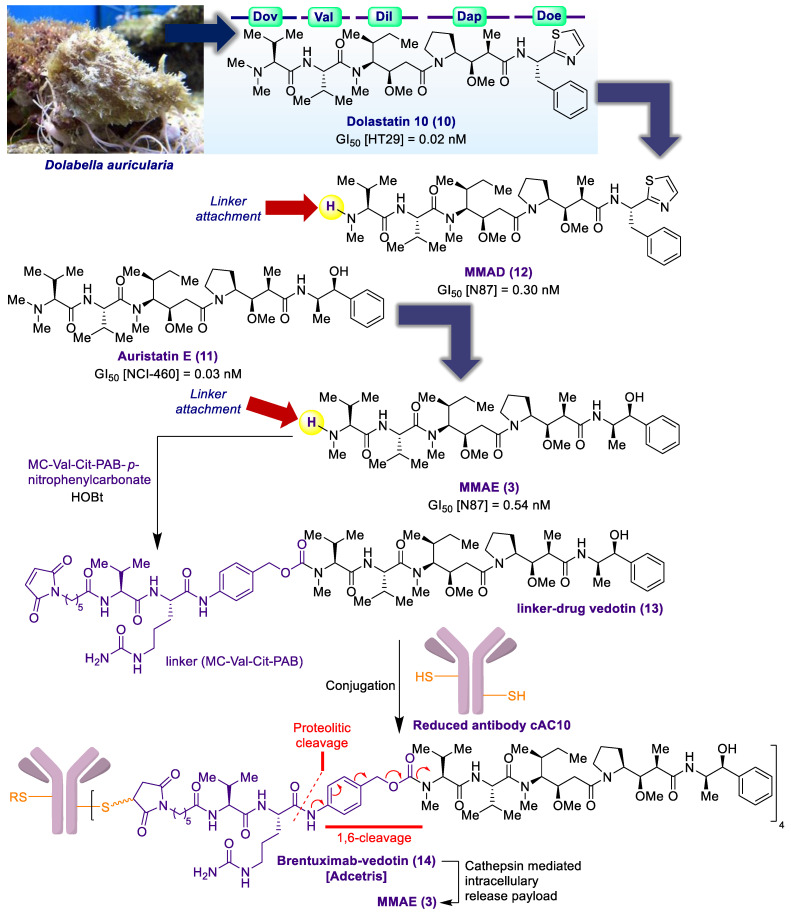
Synthesis of brentuximab vedotin **(Adcetris)** (**14**).

To this aim, the monomethyl derivative of auristatin E (**3**) was selected for the linkage of cAC10, a CD30^+^-specific antibody, through a valine-citruline dipeptide linker, flanked by a maleimidocaproyl (mc) attachment group and a *p*-aminobenzylcarbamate (PABC) spacer, which is susceptible to the action of cathepsin B proteases and subsequent 1,6-elimination to release free monomethyl auristatin E (**3**). The resulting ADC (Brentuximab vedotin, **14**), prepared by conjugation of the linker-drug mc-Val-Cit-PABC-MMAE (**13**), named vedotin, with a reduced cAC10 antibody, presented a drug-to-antibody ratio (DAR) of 4 (Scheme 1). The reduced antibody was obtained by cleavage of the interchain disulfide groups to thiols by treatment with dithiothreitol (DTT). This conjugate was highly active against CD30^+^ Hodgkin lymphoma and anaplastic large-cell lymphoma in the 3–50 pM range [72]. 

On the other hand, **14** proved to be highly stable in plasma, with a 14-day half-life in mice and was advanced to several phase I clinical trials in patients with CD30^+^ malignancies. Promising results of these trials led **14** to a phase II clinical trial in patients affected by a relapsed refractory Hodgkin lymphoma or by an anaplastic large-cell lymphoma (ALCL). Because of the excellent results obtained from these studies, brentuximab vedotin **14** was approved by the FDA in 2011 with the name Adcetris. Having demonstrated the validity of MMAE (**3**) as a payload, in the form of the linker-drug vedotin (**13**), more than 20 MMAE-based conjugates have been prepared by conjugation of **13** with other antibodies and biologically evaluated. In fact, auristatin-based payloads have become one of the most used toxins, together with maytansine and calicheamicin, in the clinical development of ADCs, and several excellent reviews have been recently reported [73,74]. Therefore, in this section of the review, only the most relevant aspects of the ADCs based on the auristatins and recent contributions not included in the prior reviews will be outlined. 

**Figure 5:**
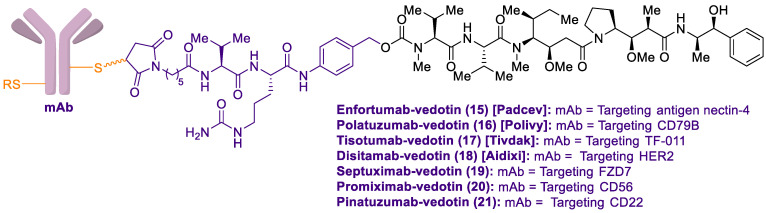
ADCs based on the monomethyl auristatin E (**3**).

Selected ADCs with vedotin currently approved or under clinical trials are the following [75,76]: (1) Enfortumab vedotin (**15**), whose antibody targets cells expressing antigen nectin-4, was approved in 2019 as Padcev for the treatment of adult patients with locally advanced or metastatic urothelial cancer [14]; (2) Polatuzumab vedotin (**16**), whose antibody targets cells expressing CD79B, was also approved in 2019 as Polivy for the treatment of large B-cell lymphoma [13]; (3) Tisotumab vedotin (**17**) [19], whose antibody targets cells expressing tissue factor TF-011, was approved as Tivdak in 2021 for the treatment of women with recurrent or metastatic cervical cancer and is also being investigated as a monotherapy in other solid tumors such as lung, colorectal, pancreatic, head and neck cancers [77]; (4) Disitamab-vedotin (**18**), whose antibody targets cells expressing human epidermal growth factor receptor 2 (HER2), was approved as Aidixi in China in 2021 for the treatment of patients with HER2-overexpressing locally advanced or metastatic gastric cancer [78]; (5) Septuximab-vedotin (**19**), whose mAb corresponds to a chimeric human-mouse IgG1 antibody that targets human FZD7, and has been evaluated in vitro and in vivo against ovarian cells, induces regression of ovarian tumor xenografts in murine models [79]; (6) Promiximab-vedotin (**20**), whose antibody corresponds to a chimeric hIgG1 that targets CD56, and was evaluated in vitro and in vivo against CD56-expressing small cell lung cancer (SCLC) cell lines NCI-H69 and NCI-H526 xenograft mouse model [80]; and (7) Pinatuzumab-vedotin (**21**), whose antibody targets cells expressing CD22, and is under phase II clinical trials for the treatment of Non-Hodgkin lymphoma [81] (Figure 5). Other vedotin-based ADCs are currently under clinical trials, such as ladiratuzumab vedotin [82], telisotuzumab vedotin [83], lifastuzumab vedotin [84], PSMA ADC [85], TAK-264 [86], or MRG002 [87], which target LIV-1, MET, NaPi2b, PSMA, GCC and HER2 receptors, respectively. On the other hand, other ADCs of this class are in preclinical stages, such as the cases of the ADCs with the antibodies SCT-200 [88], a fully humanized anti-epidermal growth factor receptor (EGFR), or MCDT2219A, an anti-CD22 monoclonal IgG1 antibody [89]. These examples, like many others, are an indication of the tremendous interest in this therapeutic strategy against cancer, resulting in a flurry of activity directed toward the development of ADCs with auristatins as payloads within the relatively short time since the approval of Adcetris.

**Figure 6:**
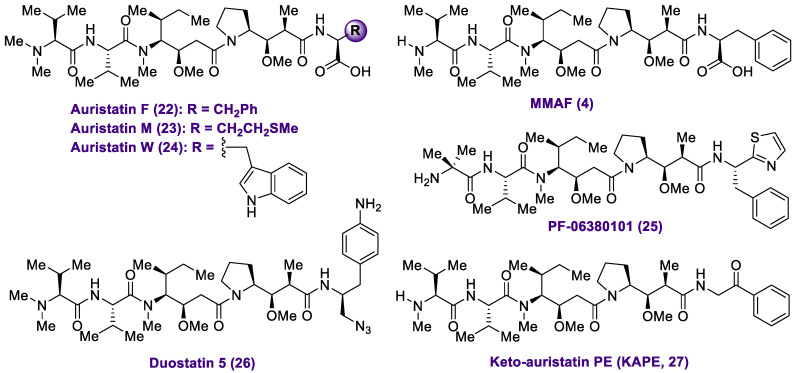
Molecular structures of new payloads based on the auristatins.

**Figure 7:**
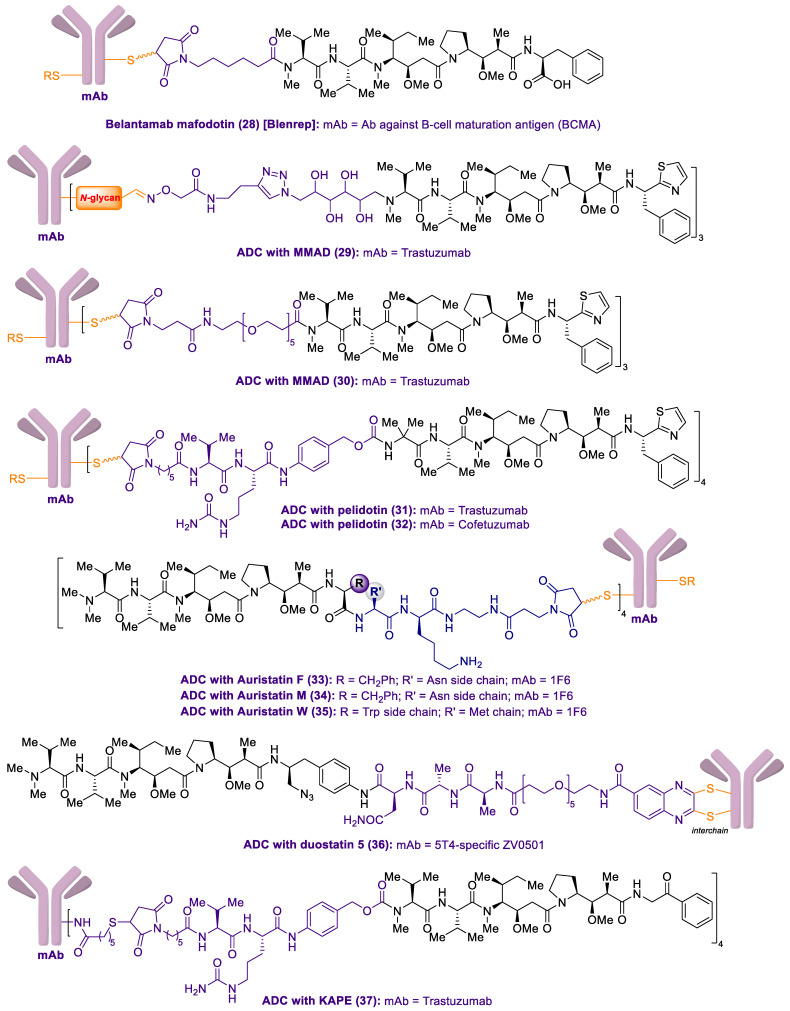
ADCs based on the auristatin payloads **4**, **22**–**27**.

Related to monomethyl auristatin E (**3**), other auristatin analogs have been selected and used as alternative payloads for other ADCs such as auristatins F (**22**), M (**23**), and W (**24**), the monomethyl analog of auristatin F (MMAF, **4**), PF-06380101 (**25**), duostatin 5 (**26**) or the keto auristatin PE (KAPE, **27**) (Figure 6). In the case of monomethyl auristatin F (MMAF, **4)**, it highlights the belantamab mafodotin ADC (**28**), which was approved by the FDA in 2020 as Blenrep for the treatment of adult patients with relapsed or refractory myeloma [17]. Another mafodotin-based ADC is depatuxizumab mafodotin, whose antibody (mAb 806) targets a unique tumor-specific epitope of EGFR and is currently being employed in several clinical trials in patients with glioblastoma or with EGFR-overexpressed solid tumors [90]. On the other hand, an antibody extensively employed in directed therapies is trastuzumab (HER2), which was extended to an ADC based on dolastatin 10 or related analogs as payloads. One example is the report by Satomaa et al., who prepared ADC **29**, which consisted of the payload MMAD (**12**) linked to the antibody through an imine group formed between an aldehyde group of the glycan portion of the glycoprotein and an amino group of the linker-drug (ADC **29**) [91]. A related ADC, based on MMAD as a payload and trastuzumab as mAb, was achieved by Yang et al. by means of a maleimide-based linker containing a polyethylene glycol [92]. The resulting ADC **30** was evaluated in a murine HER2+ ovarian SKOV3 xenograft tumor model displaying lower tumor efficacy compared with other related ADCs carrying MMAE (Figure 7).

The promising pharmacokinetic and ADME properties of the auristatin analog **25** encouraged several research groups to explore its potential as a new payload for ADC design. In fact, the conjugation with trastuzumab and the antiprotein tyrosine kinase antibody cofetuzumab, via the cleavable mc-Val-Cit-PABC linker, led to the corresponding ADCs **31** and **32**, with a DAR of 4. Thus, the anti-HER2 antibody conjugate **31** showed a strong in vivo efficacy against HER2-expressing breast, gastric, and lung murine tumor models at doses of 3–6 mg/kg, including tumors resistant to trastuzumab-DM1 [93]. The promising results led to a subsequent phase I clinical trials, which were conducted with no observation of relevant adverse effects. Similarly, clinical trials were also carried out with the cofetuzumab pelidotin **32** with patients affected by solid tumors such as platinum-resistant ovarian cancer, non-small-cell lung cancer (NSCLC), or triple-negative breast cancer. The response rates in these treatments with multiple doses of 2.8 mg/kg of **32**, administered intravenously every 3 weeks, were 27%, 19%, and 21%, respectively [94] (Figure 7). Other ADCs based on **25** are PF-06664178 and PF-06650808, which are in phase I clinical trials in patients with advanced or metastatic solid tumors [95,96].

Interestingly, for the cases of auristatin F (**22**) and the related analogs M (**23**) and W (**24**), their molecular structures were amenable to a different strategy for the design of new ADCs, allowing linkage of the antibodies at the C-terminal position, via the free carboxyl group. To this aim, auristatins F, M, and W were conjugated to the CD70 antigen-specific 1F6 antibody through a dipeptide-maleimide linker with a DAR of 4. The resulting ADCs **33**–**35** showed potent in vitro activities and maximum tolerated doses (MTDs) of 100 mg/kg, much less toxic in mice compared to the 1F6-Val-Cit-PABC-MMAF bioconjugate [97]. Given the lower toxicity exhibited by the C-linked mAbs, further conjugates were evaluated, such as the ADC derivative **36** consisting of a 5T4 antigen targeting antibody with duostatin 5 (**26**) as payload [98]. In this case, two cysteine residues of the protein were added to C-2,3 of the quinoxaline residue. The resulting ADC was more than 10 times less cytotoxic than the corresponding 5T4-specific mAb ZV0501 conjugated with MMAF, although its in vivo efficacy in a murine xenograft model did not prove to be much better against breast and pancreatic cancers. Nevertheless, several ADCs based on duostatin 5 (**26**) combined with trastuzumab and anti-CD38 antibodies are currently in phase I/II clinical trials. Other C-terminal-modified analogs of auristatin have been explored as new payloads, for example, the monomethyl auristatin PE derivatives developed by Park et al., where the keto auristatin PE derivative **27** was prepared and elaborated for the conjugation to the trastuzumab lysine via an amide bond formation [99]. This ADC **37** presented a DAR of 4.2 and showed an EC_50_ of 4.77 ng/mL and in vivo efficacy against the HER2+ murine BT-4747 xenograft model at a 1.25–5 mg /kg dose (Figure 7). 

**Scheme 2:**
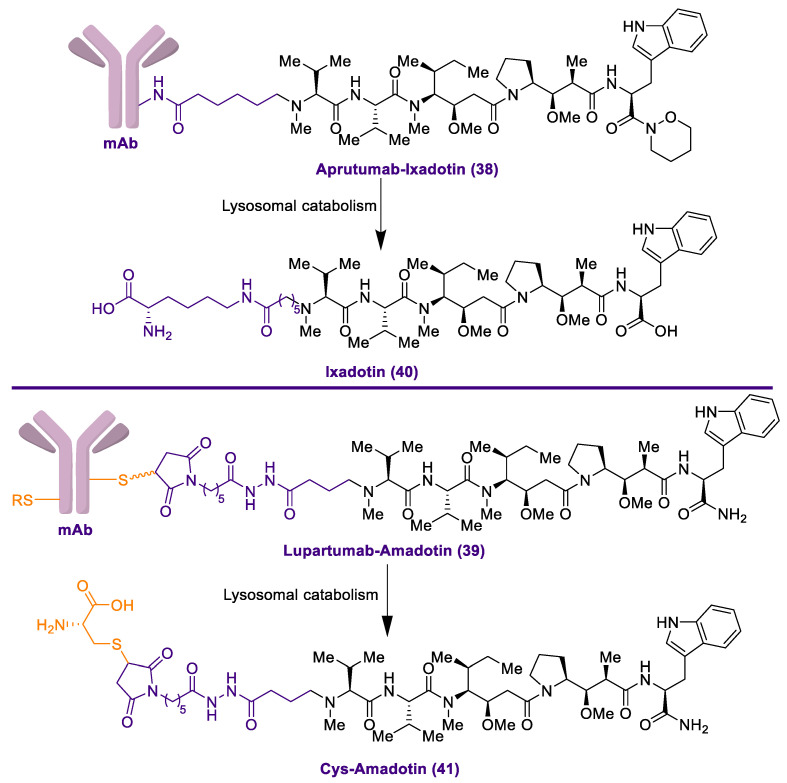
Noncleavable ADCs based on the auristatin payloads.

Inspired by these studies, Lerchen et al. have described extensive SAR studies of monomethyl auristatin analogs resulting in the identification of highly potent amide analogs, which were prepared as potential payloads for the incorporation into antibodies [100]. Particularly interesting are the ADCs **38** and **39**, corresponding to the aprutumab-ixadotin and lupartumab-amadotin, in which the linkers are not cleaved once the ADC is internalized in the cells. Thus, in the case of aprutumab-ixadotin, in which the antibody aprutumab corresponds to an FGFR2 antibody, the metabolite released ixadotin (**40**) displayed strong tumor regression in various cancers at a 5–10 mg/kg dose. However, after entering a phase I clinical trial, this was discontinued due to severe toxicities issues. For the case of lupartumab-amadotin **39**, the antibody is specific to the C4.4A antigen expressed in NSCLC and, as in the previous case, after internalization into cells, the toxin released is cys-amadotin **41**, which exhibited a significantly better efficacy against NSCLC compared to cisplatin. Similar to aprutumab-ixadotin, after entering a phase I clinical trial, the ADC was discontinued (Scheme 2).

**Scheme 3:**
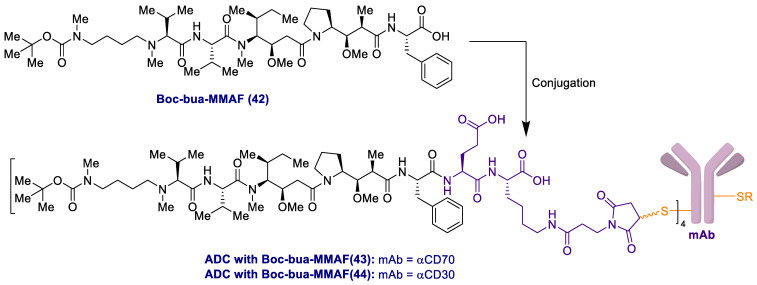
Synthesis of ADCs based on the payload **42**.

On the other hand, in contrast to MMAE (**3**), which belongs to the highly cell-permeable group, monomethyl auristatin F (**4**) is negatively charged, which renders it a payload with low cell membrane permeability. In order to increase the cell permeability of auristatin F derivatives, a series of *N*-alkylated hydrophobic MMAF analogs were synthesized, which provided highly potent analogs as free drugs with IC_50_ values in the range of 1–6 nM against colorectal and melanoma cell lines, which is in the intermediate range between MMAF and MMAE [101]. Among them, the derivative **42** was selected as a payload, being conjugated to *α*CD70 and *α*CD30 antibodies through an Asp-Lys-maleimide link. The corresponding ADCs **43** and **44** proved to be potent antitumor bioconjugates against different CD70^+^ and CD30^+^ expressing cancer cells (Scheme 3). However, these MMAF-based ADCs showed lower activity compared to the corresponding ADC counterparts with MMAE as the payload. In contrast, they showed a 2-fold better MTD (30 vs. 15 mg/kg) and reduced dose-limiting toxicity (Scheme 3). 

**Scheme 4:**
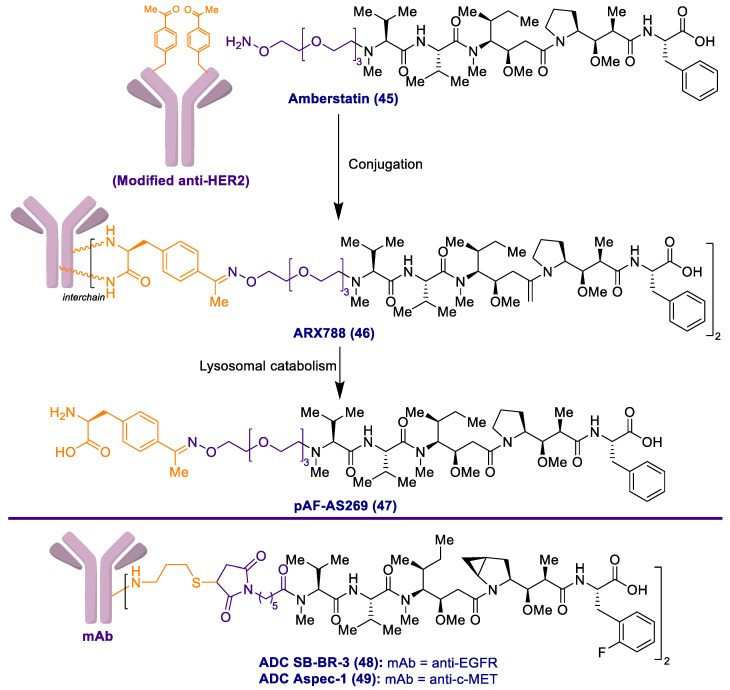
Synthesis of ADCs based on the payload amberstatin (**45**) and fluoro cyclopropyl auristatins.

Another interesting MMAF analog is amberstatin **45**, which consists of an *N*-terminal PEGylated MMAF derivative with a terminal oxyamine group, which was employed as a connection point with the antibody [102]. To this aim, two residues of *p*-acetylphenylalanine were incorporated into an anti-HER2 antibody and connected to the payload through a highly stable oxime, presenting a DAR of 1.9. This ADC **46** also corresponds to the noncleavable class, so upon cellular internalization in HER2+ cancer cells, the metabolite pAF-AS269 **47** was produced. This ADC displayed potent antitumor activities against breast, lung, gastric and ovarian cell lines with IC_50_ values in a 0.025–0.316 nM range. This strong activity was observed when this ADC was dosed in xenograft mouse tumor models possessing either high or low HER2+ expressing tumors. Together with these promising results, its high stability in serum and long half-life of 12.5 days triggered its entry into several phase I clinical trials, which are currently ongoing [103]. Another MMAF analog considered for ADC development was a cyclopropyl fluoro derivative, which was incorporated into the ADCs **48** and **49** in an attempt to address some pharmacological issues such as toxicity, expecting that the payload would not be subject to cellular efflux and thus exert less toxicity. Thus, the ADCs, consisting of the anti-EGFR and of the anti-c-MET antibodies conjugates, were prepared, revealing a DAR of 2 [104]. While the first was not well tolerated in preclinical studies at 10 mg/kg doses, the second displayed a better pharmacological profile, combined with strong cytotoxic activity against c-MET-expressing cell lines, including NCI-H1993, MKN-45, and HCCLM3 with IC_50_ values of 16.3, 7.8 and 3.2 ng/mL, respectively [105]. Surprisingly, in contrast to the strong antitumor activities of this ADC, the corresponding payload displayed weak activity against the cell lines with IC_50_ values in the range of 1700 nM. Additionally, this ADC exhibited strongly in vivo efficacy in a murine xenograft model in a c-MET-expressing pancreatic cell line (Aspc-1) at 10 mg/kg and is currently being evaluated in two phase I clinical trials in patients with advanced solid tumors (Scheme 4).

**Scheme 5:**
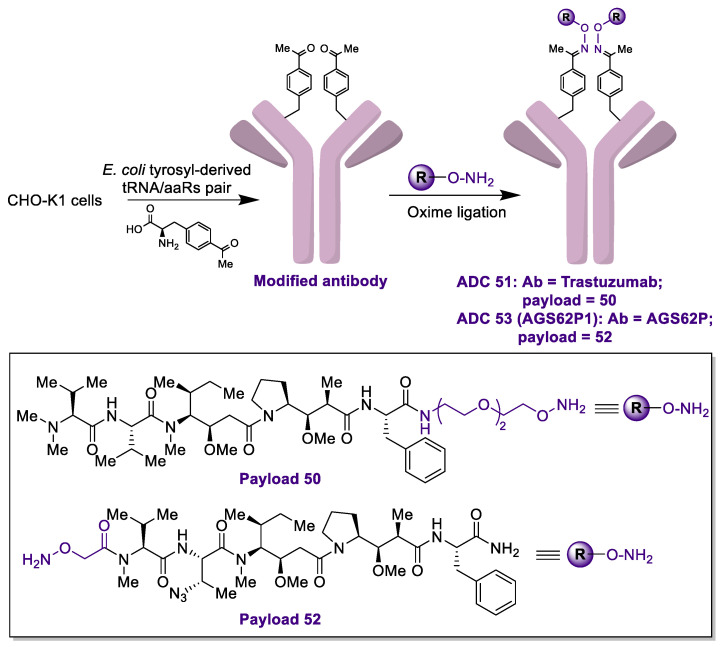
Syntheses of ADCs **51** and **53** based on oxime linkages.

Some of the most important and general concerns about ADCs are the DAR homogeneity, the presence of unconjugated mAbs, and the stability toward deconjugation. In this sense, despite the extensive use of the maleimide motif for the conjugation process, this conjugation strategy presents important drawbacks that directly affect the biological efficacy of the resulting ADC and contributes to its off-target toxicities [106]. Thus, the ADCs based on the maleimide motifs may undergo a retro-Michael reaction that leads to the loss of the linker-payload, which in turn can be transferred to circulating free thiol-containing molecules or proteins [107]. Consequently, alternative linker strategies have been explored, and some examples have been described above. With the aim of moving forward in the control and definition in a precise manner of the site and stoichiometry of the conjugates, Schultz et al. produced genetically modified trastuzumab from Chinese master ovary cells (CHO-K1), incorporating the unnatural amino acid *p*-acetylphenylalanine (pAF) into the Fab fragments of the antibody. Isolation of the modified trastuzumab, the auristatin F derivative **50**, which contains a noncleavable ethylene glycol linker with an alkoxy-amine group, was coupled to the pAF containing anti-Her2 Fab to obtain the resulting ADC **51** via a highly stable oxime linkage. This new conjugate was tested on HER2-expressing breast cancer cells (MDA-MB-435 and SK-BR-3 cancer cell lines), displaying potency and selective cytotoxicity with EC_50_ values in the range of 100–400 pM. In xenograft models, MDA-MB-435/Her2^+^ tumors were completely cleared in response to a single dose of 5 mg/kg of **51** within 14 days and did not exhibit any effect on Her2^−^ MDA-MB-435 tumors. Furthermore, **51** presented an excellent and improved pharmacokinetic profile compared with nonspecifically conjugated ADCs in terms of potency, selectivity, and stability in serum [108]. Based on this strategy, the ADC **53**, named AGS62P1, was developed, containing the monomethyl azido amide derivative **52** as payload and the antibody AGS62P, which targets FMS-like tyrosine kinase-3 (FLT3) receptor modified. This ADC was advanced to phase I clinical development for acute myelogenous leukemia, but it was discontinued due to lack of efficacy [109] (Scheme 5). 

**Scheme 6:**
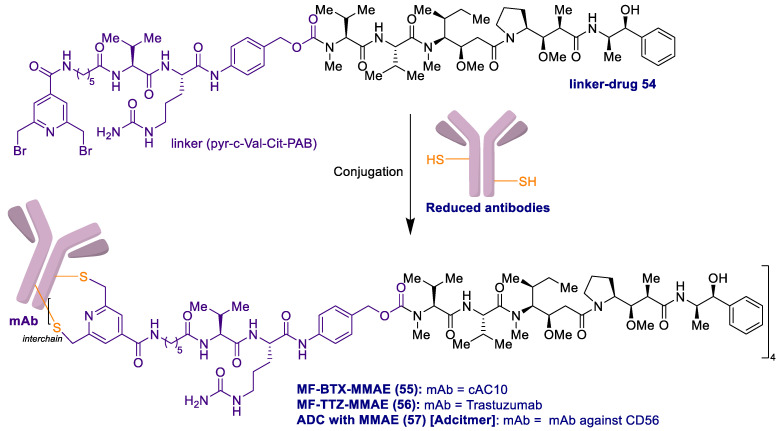
Synthesis of ADCs **55**–**57** based on the McSAF Inside Technology.

Additional technology has been reported by Juen, Martin, and coworkers, who developed the McSAF Inside Technology based on a trifunctionalized di(bromomethyl)pyridine scaffold capable of bridging the antibody chains at reduced disulfide interchain bonds to incorporate the corresponding linker-drug complex [110,111]. This new strategy allows the control of the DAR and position of the linker-payload resulting in the formation of highly stable and homogeneous ADCs. Accordingly, the authors initially prepared the ADC with the antibody brentuximab and compared it with the brentuximab vedotin (Adcetris) as proof of the concept of this new strategy. Then, after ligation of the linker-drug derivative **54**, based on the monomethyl auristatin E (**3**)**,** with the reduced anti-CD30 chimeric immunoglobulin G subclass cAC10 mAb, furnished ADC **55**, whose characterization led to a stable and homogeneous DAR distribution of 4, and excellent stability in the presence of thiol-containing proteins, such as human serum albumin (HSA), and a similar efficacy profile to Adcetris in a Karpas 299 xenograft model of CD30-positive lymphoma, with complete tumor regression in all mice when treated once at 1 mg/kg of **55 [112]**. Encouraged by these results, the authors extended their technology to other antibodies such as trastuzumab (ADC **56**) and a CD56-targeting antibody (ADC **57**), which was called Adcitmer [113]. In the case of **56**, the in vivo evaluation in a BT-474 xenograft mice model of HER2^+^ breast cancer confirmed its efficacy with complete tumor regression in all mice treated only twice at 5 mg/kg, resulting in more efficacy than the ADC ado-trastuzumab emtansine (T-DM1), which only cured three out of eight mice. For **57**, its antitumor properties were evaluated against Merkel cell carcinoma (MCC), which is a rare and aggressive cancer of the skin that expresses CD56 proteins. Thus, **57** was found to be cytotoxic on MCC cell lines with IC_50_ values in the range of 2.5–30.7 nM. On the other hand, in an MCC xenograft mouse model, it was found that **57** significantly reduced tumor growth with no signals of toxicity effects (Scheme 6).

**Scheme 7:**
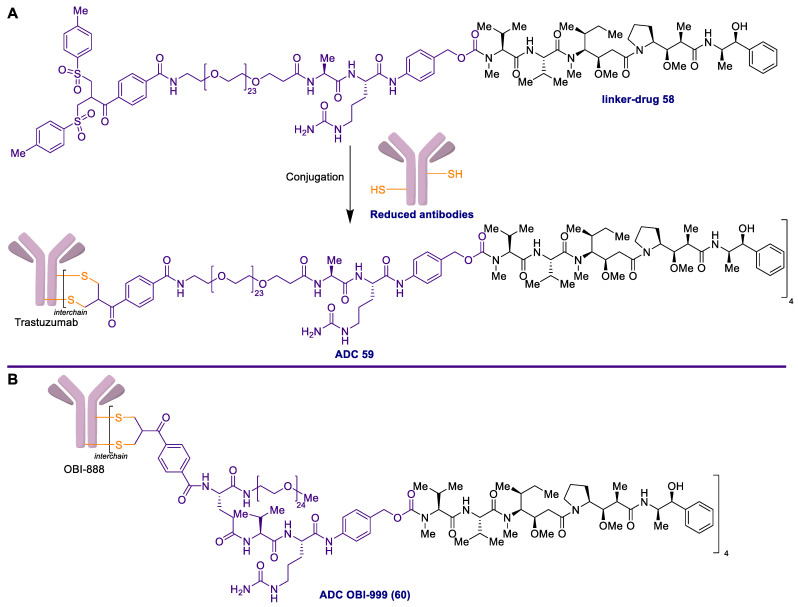
Synthesis of ADCs **59** (**A**) and **60** (**B**) based on a bis-alkylation process of the antibody.

A related strategy was delineated by Godwin et al., who employed the linker-drug **58** that carries a reactive bis-sulfone moiety capable of reacting with the cysteine sulfur atoms of the protein to undergo a bis-alkylation process [114]. This reaction resulted in a covalent rebridging of the interchain disulfide bonds of the antibody, leaving the protein structurally intact. The resulting ADC **59**, in which the antibody was trastuzumab, presented a well-defined DAR of 4 and displayed excellent stability properties and antitumor activities against HER2 expressing cancer cells [115] (Scheme 7A). This assembly technology was applied in the preparation of the ADC OBI-999 (**60**), which contains the antibody OBI-888 that targets the tumor-associated carbohydrate antigen globo H (GH). OBI-999 exhibited very suitable pharmacological properties, including efficient internalization rate, tumor-specific ADC accumulation, payload released against GH-expressing cells, a bystander effect, efficacy in animal models, and a safety margin in monkeys [116] (Scheme 7B).

**Figure 8:**
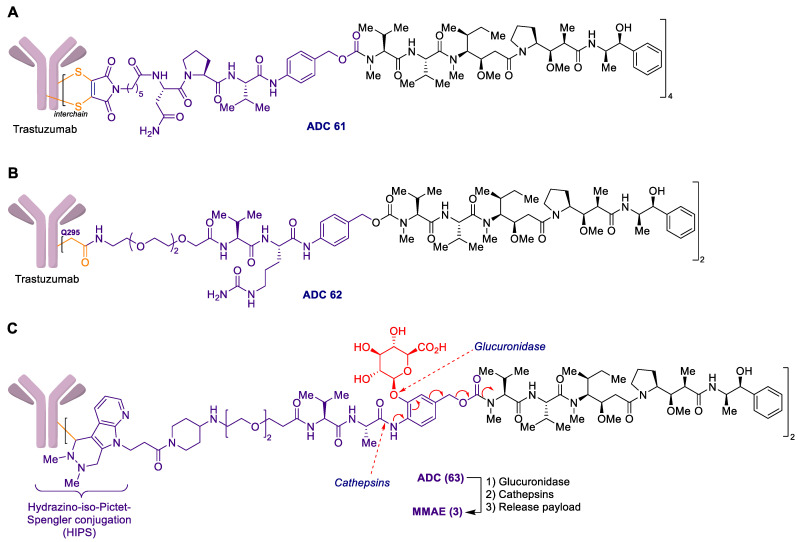
Structures of the ADCs **61**–**63** (**A**–**C**).

An interesting strategy was developed for the preparation of the ADC **61** [117], in which the authors introduced a tripeptide (Asn-Pro-Val) in the linker domain, which was recognized as a specific substrate of human neutrophil elastase (HNE), an enzyme overexpressed in tumor microenvironments. The resulting HNE-sensitive conjugate was able to kill antigen-positive cancer cells with IC_50_ values in the 0.23–0.36 nM range and no cytotoxic activity on antigen-negative cells. However, in these later cases, when exogenous HNE was added (0.15 μM), the antitumor activity was restored in the nM range, suggesting an extracellular release of the drug and a subsequent bystander killing effect. Although it remains to be confirmed the efficacy of **61** in in vivo studies, the incorporation of this peptide could be applied in the design of new ADCs with a dual intra- and extracellular mechanism of drug release that could affect a large variety of tumor types because of the protease action of HNE, whose levels are high in many cancers (Figure 8).

An additional interesting case is represented by the ADC **62**, in which the authors produced a steady and homogeneous ADC with a DAR of 2 via specific assembly of glutamine-295 of trastuzumab with the linker-MMAE by the enzymatic action of microbial transglutaminase. The ADC **62** exhibited remarkable antitumor activity against HER2-positive cancer cells in xenograft models, high serum stability, and an excellent PK profile [118] (Figure 8). A closely related enzymatic strategy for a site-specific conjugation in the preparation of homogeneous ADCs was also employed for MMAE as payload by use of formylglycine-generating enzymes [119]. As mentioned above, a common side-effect of vcMMAE-conjugated ADCs is the off-target toxicity as a result of the premature cleavage of the Val-Cit peptide by the effect of extracellular peptidases. Among the different approaches to surmount this serious limitation, Drake et al. envisioned a possible linker requiring a tandem enzymatic action in which the second cleavage is hindered until the first one occurs [120]. This cleavage requirement would limit the loss of the payload during circulation, reducing off-target toxicities. To this aim, they introduced in the linker domain a β-glucuronide moiety as a temporary hydrophilic protecting group for the dipeptide linker. The appended monosaccharide unit would be stable during circulation in the bloodstream but cleaved upon internalization by the action of glucuronidases, lysosomal enzymes often upregulated in malignant cells. Subsequently, after the removal of the monosaccharide, the peptide would be available for further processing with the subsequent release of the payload. The corresponding ADC developed by the authors, ADC **63**, contained an anti-CD79b antibody that was linked to the payload through a hydrazine-iso-Pictet-Spengler (HIPS) conjugation system [121] (Figure 8). The resulting ADC proved to be stable in circulation, well tolerated in toxicity studies in rats, and excellent antitumor activity in xenograft tumor models. 

**Figure 9:**
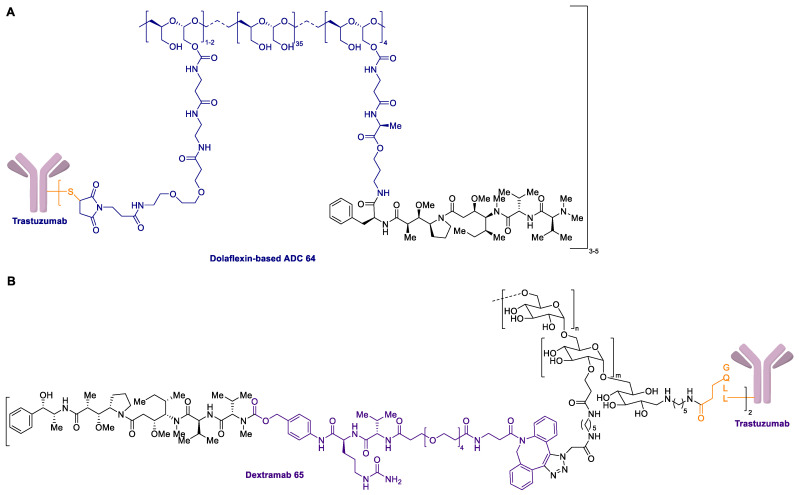
ADCs **64** and **65** based on the new platforms (**A**) dolaflexin and (**B**) dextran.

As with this case, we can find in the literature many other examples in which auristatins are employed as payloads of choice as a benchmark for the development of new strategies and tactics in the field of ADC with the possibility to be expanded to other payloads. Remarkable examples are the development of novel platforms that allow high drug loading and a controlled bystander effect. Such is the case of dolaflexin, also known as fleximer, a novel auristatin ADC platform consisting of a polymer of poly-1-hydroxymethylethylene hydroxymethylformal, which provides high hydrophilicity and polyvalence with DARs of 10–15 [122]. The resulting ADC **64**, with trastuzumab as an antibody, displayed an enhanced activity relative to conventional ADCs and increased tolerability with minimum toxicity (Figure 9A). Clinical studies of the dolaflexin-based ADC in the context of XMT-1536, which targets NaPi2b, were considered according to the promising results obtained with the ADC XMT-1536 against MSCLC adenocarcinoma and epithelial ovarian cancer in animals [123]. In the same strategic direction, dextran polysaccharide was also employed as a DAR- and polarity-enhancing scaffold. In this case, the functionalized dextran derivative was coupled with an engineer-modified trastuzumab, possessing a microbial transglutaminase (mTG) recognition tag LLQG at the C-termini of its heavy chains, and then assembled with a DBCO derivative of MMAE through a strain-promoted azide-alkyne cycloaddition (SPAAC) with the azide groups previously introduced in the polysaccharide. The resulting ADC **65**, named dextramabs, presented a DAR of 8 without compromising stability and solubility properties [124]. In addition, the ADC targeted and killed HER2-positive SK-BR-3 cells at subnanomolar concentrations (Figure 9B). Similarly novel and interesting is the poly-ADP-ribose polymer employed in the design of new ADCs for the enhancement of their physicochemical and pharmacological properties, in which a DBCO-MMAF derivative was employed [125].

**Scheme 8:**
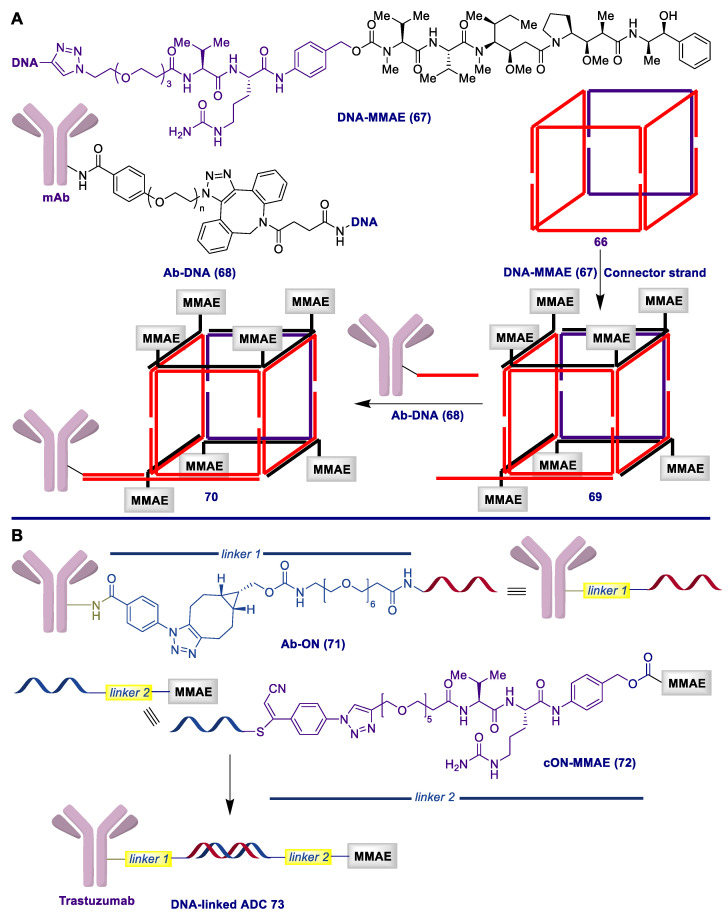
(**A**) Synthesis of ADC integrated into a wireframe DNA cube. (**B**) Synthesis of a DNA-linked ADC.

Finally, Gothelf et al. have designed an interesting ADC based on the use of a DNA nanostructure that would allow for high loading of the drug and exquisite control of DAR, resulting in homogeneous and well-defined ADCs, key features required for their clinical efficiency and safety [126]. The programmability of oligonucleotides should allow for the precise design of DNA-based frameworks with suitable modifications for a controlled and precise arrangement of the drug in a predefined ratio. Inspired by the pioneering works of Sleiman et al. in the construction of 3D-DNA nanostructures [127], the authors prepared a DNA wireframe cube from eight single-stranded domains, of which seven were bound to complementary strands conjugated to the MMAE payload, while the eighth stranded domain is used for connection to the antibody. The resulting cube **66** presented a diameter of approximately 15 nm containing four single strands of 90 bases. For the preparation of the MMAE-containing strand, the azide derivative of the payload was reacted with an alkyne-modified DNA strand in the presence of Cu(I) to afford the DNA-MMAE conjugate **67**. These strands were then successfully assembled to the wireframe DNA cube to obtain complex DNA-MMAE **69**. Prior to the assembly of the cube DNA-MMAE **69** with the complex Ab-DNA, the authors introduced a connector strand in the DNA wireframe cube since direct binding of the antibody DNA construct to the remaining single chain on the cube would be problematic due to steric hindrances. For the preparation of the antibody DNA fragment, trastuzumab was chosen as the antibody, and the linkage to the DNA was achieved by the use of a lysine-directed labeling reagent (LDLR), which allowed the easy introduction of an azide group, which reacted with the dibenzocyclooctyne-modified DNA strand (DNA-DBCO) to deliver Ab-DNA **68**. Incubation at room temperature of the Ab-DNA **68** with **69** afforded the complex DNA-ADC **70**, which was fully characterized (Scheme 8A). 

The ADC was evaluated against SKBR3 and A431 cancer cell lines, confirming a decrease in cell viability only on the HER2-expressing SKBR3 cell line after exposure to the ADC over several days. In addition, the evaluation of its stability was accomplished by exposure of **70** to serum, proving that after a 24 h period, the structure was completely degraded. While this approach provided proof of concept of this new strategy for the design and preparation of new ADCs, several issues still need to be addressed prior to its use for in vivo studies. In this sense, one possibility to be explored is the replacement of nucleotides by nucleotide analogs capable of resisting enzymatic degradation, which is the reason for its low stability and subsequent degradation in serum. In a related contribution, Wagner et al. prepared a DNA-linked ADC by hybridization of an antibody-ON **71** conjugate with the cON-drug conjugate **72 [128]**. The resulting ADC **73**, in which the conjugation between the antibody and the payload was achieved by assembly of the oligonucleotide strands, was surprisingly stable in human plasma. With respect to its biological properties, **73** showed potent cytotoxicity against HER2-positive cells, inducing cell death at nanomolar concentrations (EC_50_ = 1.93 nM) (Scheme 8B). 

Although it is out of the scope of the present review, it is important to mention alternative tactics that are being explored with the auristatins with the objective of targeting cancer cells in a highly selective manner by means of platforms different from antibodies recognized by tumor antigens. Among these alternative devices, it is worth highlighting low-molecular-weight ligands for antigens such as the prostate-specific membrane antigen (PSMA) [129] or integrin ανβ_3_ [130], aptamers [131], gold nanoclusters [132] or radiolabeled proteins [133].

### 2.2. Antibody-Drug Conjugate Based on Halichondrin B

**Figure 10:**
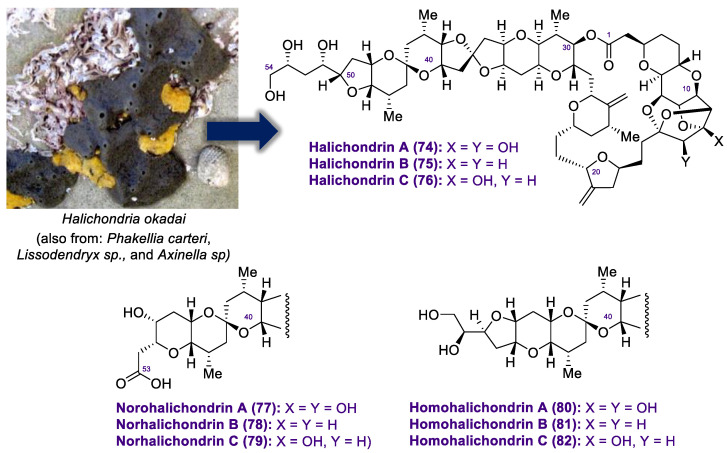
Molecular structures of the halichondrins.

The halichondrins are a family of natural products characterized by a highly complex polyether macrolide containing an unprecedented 2,6,9-trioxatricyclo[3.3.2.0]decane ring system. Halichondrins were originally isolated from the Japanese marine sponge *Halichondria okadai* [134], and further congeners were subsequently discovered in unrelated sponges such as *Phakellia carteri* [135], *Lissodendryx* sp. [136,137,138], and *Axinella* sp. [139]. Except for halichondrin A [140], all the subgroup members have been isolated from natural sources. The structure diversity that distinguishes the various members involves two sites: the oxidation state at C10, C12, and C13 of the C8–C14 polycycle and the length of the carbon backbone. According to this, the halichondrins are classified into the A–C (**74**–**76**) series or the norhalichondrin (**77**–**79**) and homohalichondrin (**80**–**82**) series (Figure 10).

Among all the related congeners of *Halichondria okadai* metabolites, halichondrin B (**75**) was identified as the most prolific and important member based on its impressive biological profile. Halichondrin B showed high potency (IC_50_ of 0.093 ng/mL) against B-16 melanoma cells in vitro, and further assays indicated potent antiproliferative effects on several cancer cell lines at nanomolar concentrations [139,141], which was translated to in vivo studies by increasing survival times in mice models of melanoma and leukemia [142]. Halichondrin B mode of action is based on an interaction of the natural product with tubulin. In this way, nonproductive tubulin aggregates are formed, and the assembly of microtubules is suppressed without effect on microtubule shortening. Thus, this leads to arrest in the G2-M phase of the cell cycle and apoptosis, resulting in cell death following prolonged mitotic obstruction [143,144]. Studies of its mechanism of action revealed differences from those of other antimitotic compounds, and the activity was also observed on cancer cells resistant to other antimicrotubule agents [145].

**Figure 11:**
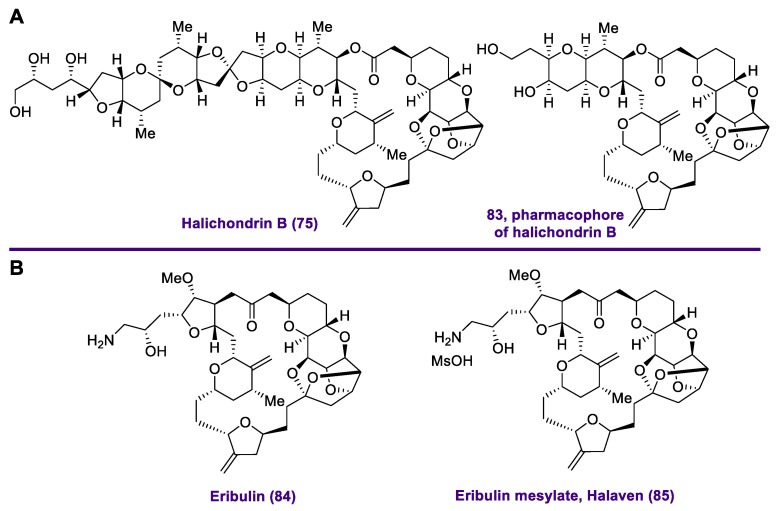
(**A**) Molecular structures of halichondrin B (**75**) and pharmacophore **83**; (**B**) Molecular structures of eribulin (**84**) and eribulin Mesylate (**85**).

The impressive molecular architecture and outstanding antitumor activities coupled with the unprecedented mechanism of action displayed by halichondrin B (**63**) prompted intense research activity directed toward its total synthesis, analog design, and biological evaluation. Initial attempts to provide material for further biological evaluations were made by isolation of halichondrin B (**75**) from related sponges; however, the extremely low yields of the isolated compound hampered the preclinical studies. This issue was solved by total synthesis, which was initially accomplished by the Kishi group in 1992 [146]. Later, both the Kishi group and scientists at Eisai carried out a campaign toward simplified analogs that culminated with the identification of the minimum pharmacophore of halichondrin B (**75**), revealing that the structural features responsible for the cytotoxicity were the right-half macrolactone fragment, represented by compound **83** [147]. Further structural and biological optimization led to the discovery of eribulin (**84**), which is structurally simpler than halichondrin B. Preclinically, eribulin showed potent activity against several human cancer cell lines in vitro and in vivo through its antimitotic effect [144,148]. In addition, eribulin showed unique effects on tubulin, causing vasculature remodeling within the tumor microenvironment and mesenchymal-to-epithelial transition of breast cancer cells [149,150]. Ultimately, eribulin mesylate **85** (Halaven) [151,152] was approved to treat metastatic breast cancer in the USA in 2010, and to date, it has been approved in approximately 70 countries (Figure 11).

**Scheme 9:**
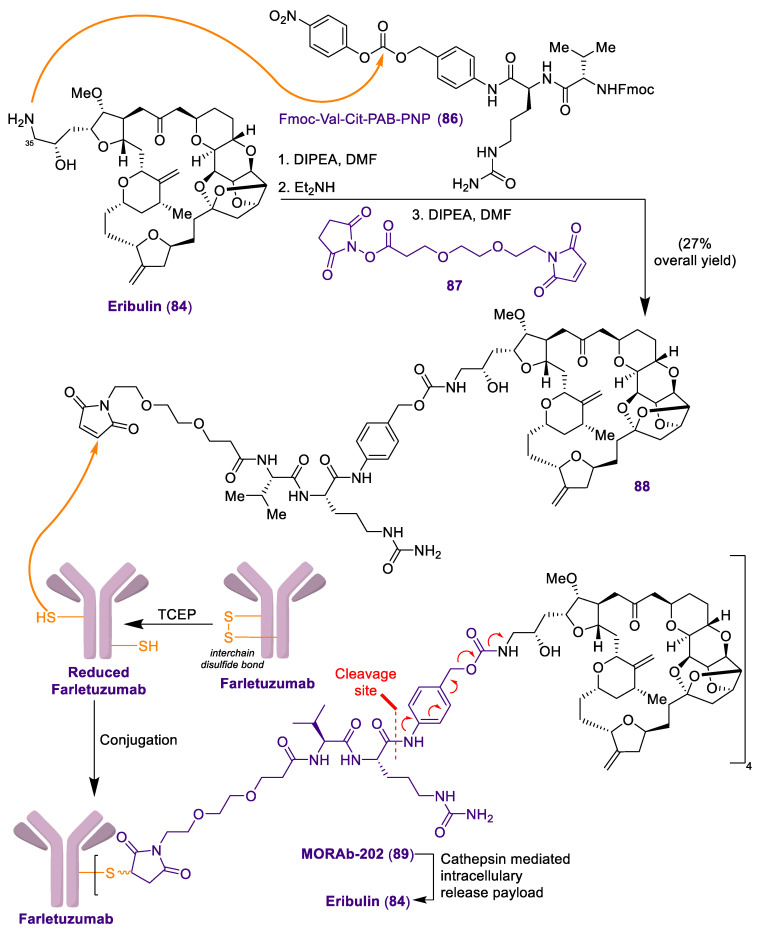
Synthesis of MORAb-202 (**89**) and intracellulary release of eribulin payload. [Val: valine; Cit: citrulline; PAB: *p*-aminobenzylcarbamyl; PNP: *p*-nitrophenol. TCEP: tris(2-carboxyethyl)phosphine].

Not surprisingly, because of these excellent and striking antitumor activities, including antimitotic and nonmitotic effects, together with its unprecedented mechanism of action, eribulin (**84**) has been targeted as a payload for ADCs. Albone et al. [153] designed, synthesized, and characterized an ADC, MORAb-002, utilizing the microtubule-targeting agent eribulin as the cytotoxic payload. The authors focused on the folate receptor alpha (FRA), which is a glycosylphosphatidylinositol (GPI)-linked protein that is frequently overexpressed in various malignant tumors of epithelial origin, including ovarian, lung, and breast cancer, while largely absent from normal tissues. To complete the ADC design, they chose farletuzumab as the humanized anti-human FRA mAb, and after optimization of the linker and conjugation approaches, the cathepsin B cleavable Val-Cit-PAB linker was selected (Scheme 9).

The connection of eribulin (**84**) and the linker was based on the nucleophilic attack of the primary amine at carbon-35 in eribulin to the carbonate group contained in Fmoc-Val-Cit-PAB-PNP (**86**). Subsequent Fmoc deprotection, using diethylamine, afforded the corresponding free amine, which was reacted with **87** to obtain the maleimide linker attached to eribulin **88**. Then, the antibodies were prepared for conjugation by rupturing their interchain disulfide moieties into thiols via reaction with tris(2-carboxyethyl)phosphine (TCEP). The resulting reduced antibody was conjugated with the linker-eribulin **88** through its maleimide moiety to furnish the desired ADC, MORAb-202 (**89**), at a drug-antibody ratio of 4.0 (Scheme 9). Ultimately, eribulin payload is released intracellularly by the action of cathepsin, a lysosomal protease highly expressed in cancer cells versus normal cells (Scheme 9).

After completion of the synthesis of the ADC **89**, the same authors carried out in vitro and in vivo studies, showing that MORAb-202 had picomolar potency on the FRA cancer cell line IGROV1, subnanomolar potency on NCI-H2110 and OVCAR-3 cells, and nanomolar potency on cells lines of moderate to low expression of FRA. In addition, MORAb-202 showed improved specificity in vitro compared with farletuzumab conjugated with other microtubule-targeting agents as payloads. In vivo studies were carried out in an NCI-H2110 model in CB17SCID mice and in PDX models of NSCLC and gastric cancer and demonstrated prolonged tumor growth inhibition in both models at a dose of 5 mg/kg [153]. Further biological studies on the same ADC were performed by Yamaoka and coworkers [154]. They characterized the effect of MORAb-202 on breast cancer and NSCLC cell lines, showing that the eribulin released into HCC1954 cells exhibited a bystander killing effect by diffusing through cell membranes into intercellular spaces and killing the neighboring MCF7 cells in HCC1954-MCF7 co-culture systems. In vivo experiments showed growth suppression of T47D and MCF7 orthotopic tumors.

These results were like those reported by Furuuchi et al., who also showed that MORAb-202 exhibited a clear in vitro bystander cytotoxic effect, and it was highly cytotoxic to FRA-positive cells in vitro, with the limited off-target killing of FRA-negative cells. In addition, in vivo toxicology studies in non-human primates suggested that the major observed toxicity is hematologic toxicity. Recently, results from a phase I first-in-human trial to evaluate the safety and efficacy of MORAb-202 were released by Shimizu et al. This study showed that MORAb-202 was well tolerated, with a mild toxicity profile. In addition, the MORAb-202 was able to have an effect on various tumors (ovarian, endometrial, triple-negative breast, and NSCLC cancers) that had relapsed after failure to respond to standard therapy. The expansion of this phase I study is currently ongoing.

### 2.3. Antibody-Drug Conjugates Based on PM050489

**Figure 12:**
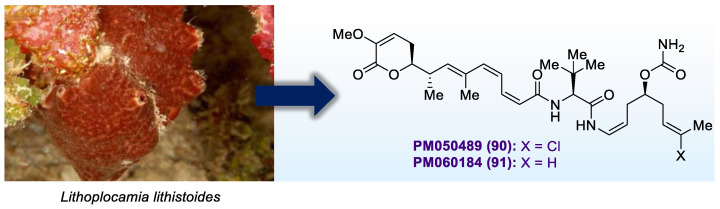
Molecular structures of PM050489 (**90**) and PM060184 (**91**).

PM050489 (**90**) and its dechlorinated analog, PM060184 (**91**), are two marine natural products isolated from the Madagascan sponge *Lithoplocamia lithistoides* by PharmaMar (Figure 12) [155]. 

They are the first members of an unprecedented new class of polyketides, structurally characterized by an α,β-unsaturated δ-lactone, a conjugated triene system, and an L-*tert*-leucine linked via a (*Z*)-enamide to a diene system containing a carbamate subunit. Both polyketides exhibited subnanomolar in vitro activity in human cancer cell lines (colon HT-29, lung A-549, and breast MDA-MD-231) and potent antimitotic activity, with an IC_50_ of 26.4 nM. Interestingly, further studies revealed that these molecules inhibit microtubule assembly via a novel mechanism of action by suppressing microtubule shortening and growing to a similar extent [156]. 

**Scheme 10:**
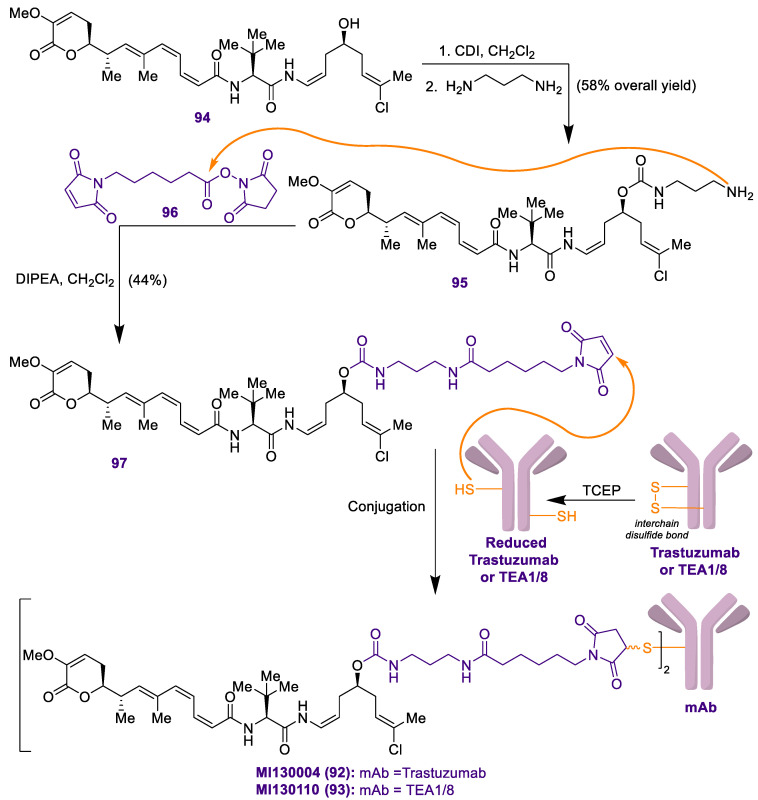
Synthesis of MI130004 (**92**) and MI130110 (**93**).

The combination of extremely high potency and the novel mechanism of microtubule dynamics impairment led PharmaMar to synthesize and characterize two ADC based on PM050489 (**90**), MI130004 (**92**), and MI130110 (**93**) [157]. MI130004 is based on the conjugation of PM050489 to cysteine residues of trastuzumab, while in MI130110, PM050489 is conjugated with an anti-CD13 TEA1/8 antibody. Both ADCs contain a maleimide-based noncleavable linker. The synthesis of both ADCs is summarized in Scheme 10. Accordingly, advanced precursor **94** was transformed into **95** in two steps, involving the reaction of the secondary alcohol in **94** with 1,1′-Carbonyldiimidazole (CDI) to obtain the corresponding carbamate, which was subsequently reacted with propane-1,3-diamine to obtain **95** in 58% overall yield. The maleimide-based linker was introduced by the nucleophilic attack of the terminal amine in **95** to the activate ester **96** to afford the maleimide linker attached to the payload **97** in 44% yield. The antibodies, trastuzumab or TEA1/8, were prepared by reduction with TCEP. The resulting reduced antibodies were then conjugated with **97**, via its maleimide moiety, to furnish MI130004 (**92**) and MI130110 (**93**), which contain two molecules of PM050489 per antibody molecule. 

With both ADCs in hand, the authors carried out in vitro and in vivo biological studies. MI130004 showed remarkable in vitro antiproliferative activity in a panel of cancer cell lines (breast, gastric, and ovary). The antimitotic activity was only detected in HER2-positive cancer cells, in contrast with the activity of its payload PM050489, which affected both HER2-positive and HER2-negative cancer cells, while MI130004 had suitable selectivity for cancer cells that overexpressed HER2. In vivo studies were carried out in mice breast, gastric and ovarian models. The results showed a tumor volume reduction together with an increase in survival only in HER-positive tested models, indicating selectivity of the ADC for HER2-expressing cells. In addition, toxicity or body weight loss was not observed at the drug doses used (up to 10 mg/kg) [157]. 

In the case of MI130110, in vitro anti-proliferative activity was selective on CD13-expressing cancer cells (HT1080, NB-4, and U-937), and it possessed the same cytotoxic effects as for PM050489. After the evidence of these antitumor effects in vitro, the authors decided to investigate the effects in vivo. MI130110 was tested in a CD13-positive fibrosarcoma murine model (HT1080 cells) and in a mouse model of myeloma cells not expressing CD13. The results showed that MI130110 exhibited excellent antitumor activity with total remission in a significant number of treated animals, but only on the model expressing CD13, thus highlighting the selectivity of the ADC to its target and its stability in circulation [158]. These results demonstrate the potential of these ADCs as promising antitumor therapeutic agents.

### 2.4. Antibody-Drug Conjugates Based on Shishijimicin A

**Figure 13:**
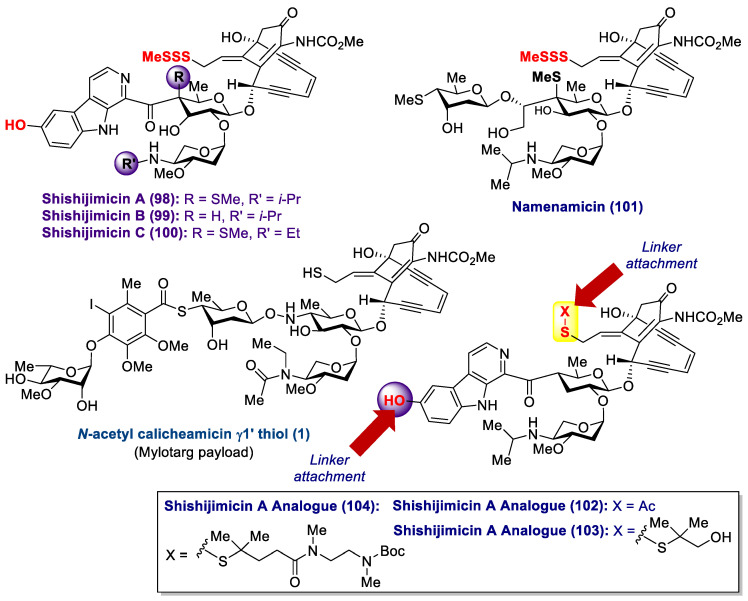
Molecular structures of the shishijimicins and the shishijimicin A analogs **102**–**104** as potential payloads.

The discovery in 2003 from the marine ascidian *Didemnum proliferum* of shishijimicin A (**98**), together with other related members (shishijimicins B (**99**), C (**100**), and namenamicin (**101**)), and the recognition of their impressive antitumor properties (IC_50_ = 0.48 pM against P388 leukemia cells) [159] prompted a flurry of research activity recently culminated with its total synthesis, analog design, and biological studies by Nicolaou et al. Taking into account the structural similarities of the shishijimicins and calicheamicins, most notably the presence of a common enediyne moiety, which is responsible for their antitumor properties, via the DNA cleavage, and drawing inspiration upon the ADCs Mylotarg and Besponsa, in which payloads correspond to *N*-acetyl calicheamicin γ_1_^I^ (**1**), the Nicolaou group decided to explore the potential of shishijimicin A-type molecules as new payloads in ADC-based cancer therapies. The seminal contributions of this group in the chemistry and biology of this fascinating natural product have consisted of the total synthesis of natural shishijimicin A (**98**) [160], SAR studies with an array of analogs [161] and studies on the biological mode of action [162]. All this work has allowed for the identification of the analog **102**, in which both the hydroxyl and methylthioether functionalities are deleted and the trisulfide moiety replaced by a thioacetate group, resulting in a very potent, structurally simplified derivative comparable to the natural product. Due to the aforementioned features, this compound was initially selected as a privileged payload, with the phenolic group of the carboline domain serving as the attachment site for the linker. On the other hand, based on the calicheamicin-based ADCs Mylotarg and Besponsa, in which the antibodies are linked through a sterically hindered disulfide structural motif, the shishijimicin analogs **103** and **104** were also chosen as suitable payloads to attach to the corresponding antibodies through linkers that could be connected either to the phenolic as well as to the disulfide domains, respectively (Figure 13). Importantly, the synthesis delineated and extensively optimized for the shishijimicins by Nicolaou et al. gave rapid and efficient access to sufficient amounts of the targeted payloads and made possible the preparation and biological evaluations of the corresponding ADCs [163]. 

**Figure 14:**
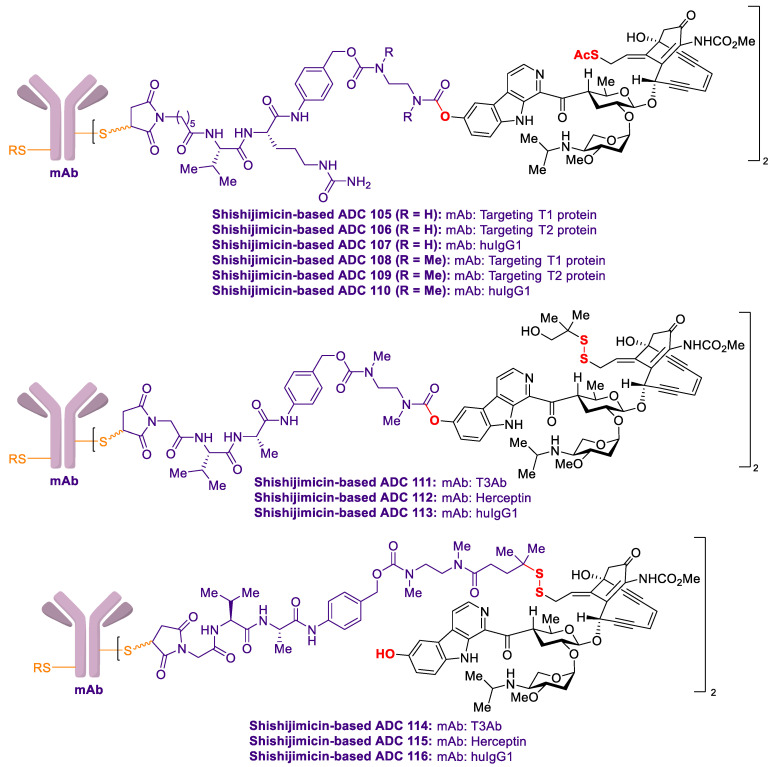
ADCs based on shishijimicin payloads.

Thus, the ADCs **105**–**110** were prepared from payload **103** by the introduction of three antibodies, including two targeting highly expressed and well internalizing cell surface proteins, T1 and T2, and the one nontargeting antibody, huIgG1, as a control. The conjugation processes were accomplished in a site-specific manner resulting in homogeneous ADCs with a DAR of 2. In contrast to the ADCs **105**–**107**, which displayed no cytotoxicity, likely due to poor stabilities and nonspecific release of the warhead, the ADCs **108**–**110** exhibited an IC_50_ value of 50.25 pM in a genetically engineered target overexpressing HEK293T cell line. For the payloads **103** and **104**, the conjugation was performed with two targeting antibodies, T3Ab and Herceptin, obtaining ADCs **111**–**116** with a DAR of 2 (Figure 14). Gratifyingly, both classes of conjugates showed excellent specific cytotoxicities in relevant cell lines, such as SKOV3 and NCI-N87, with IC_50_ values of 8.21 and 6.41 pM in the genetically engineered target 3 overexpressing HEK293T cell line. In a similar manner, the Herceptin conjugates exhibited highly specific cytotoxicity in three Her2 overexpressing cell lines (SKBR3, SKOV3, and NCI-N87). In terms of stability, the ADC **116** remained intact in mouse plasma after 7 days, while only 77% of the ADC **113** remained intact. Comparatively, with respect to the calicheamicin-based payload, the shishijimicin framework offers at least two different positions for linker attachments, and both were explored, providing interesting results that may be further investigated for the optimization of pharmacokinetic, pharmacodynamic, and stability properties in order to improve the efficacy and safety profiles.

### 2.5. Antibody-Drug Conjugates Based on Aplyronines

The aplyronines A-C were isolated from the sea hare *Aplysia kurodai*, a kind of mollusca collected on the Pacific coast of Mie Prefecture (Japan), in 1993 by Yamada et al. [164], who elucidated their molecular structures [165,166,167] and achieved their first total syntheses of aplyronine A, B and C [168]. A few years later, they isolated and characterized otheraplyronines congeners corresponding to aplyronines D–H [169,170]. Together with their complex molecular architectures, featured by the presence of a highly substituted 24-membered macrolactone core, a side chain terminating in an *N*-methyl-*N*-vinyl formamide moiety, and an amino acid residue attached to C29, the aplyronines displayed impressive antitumor activities with aplyronines A (**105**) and D (**106**) as the most active members, with cytotoxicities of 0.039 and 159 ng/mL against HeLa-S3 cells, respectively.

Karaki et al. demonstrated that aplyronine A (**105**) inhibits actin, an abundant protein in the cytoskeleton that regulates cell functions such as cell division or muscle contraction, by depolymerizing F-actin by severing it and complexing with G-acting in a 1:1 molecular ratio [171,172,173,174]. They also suggested that the activity may be due to the side chain of aplyronine A (**117**). This interaction was further demonstrated by photoaffinity labeling experiments and the isolation of a crystal structure of the actin-aplyronine A complex, confirming the binding site of aplyronine A to actin [175]. This site is composed of hydrophobic amino acids and is termed an ahydrophobic cleft, where the aliphatic chain of aplyronine A binds. The macrocycle binds to three amino acid residues of subdomain 3: the C13 methoxy group of aplyronine A with the carbonyl group of Ser145, the C9 hydroxyl group with Glu334, and the C10 methyl group with the hydrophobic pocket of Pro332 and Ser145 [176]. Some years after, Kigoshi et al. revealed that the aplyronine A mode of action is based on the inhibition of microtubule assembly by the interaction of a 1:1:1 heterotrimeric complex of aplyronine A-actin-tubuline, inhibiting the spindle formation and mitosis in cancer cells exhibiting the potential of aplyronines as antitumoral agents [177].

Studies of the structure-activity relationships against HeLa-S_3_ cells revealed that in terms of cytotoxicity, the length and the presence of the side chain are essential. The presence of an *N*,*N*,*O*-trimethylserine ester moiety located on the macrocycle and the conjugated diene is responsible for the strong cytotoxicity, as well as the two hydroxyl groups of the side chain. Either *N*,*N*-dimethylalanine or N-methylformamide groups are necessary for the strong cytotoxicity. In terms of depolymerizing activity, the length and presence of the side chain are essential, as well as the combination of the macrocycle and the side chain. The absence of the acetyl ester in the side chain proved to be important to maintaining the activity, and the presence of the *N*-methylformamide, the *N*,*N*-dimethylalanine ester, the conjugated diene moiety, and the *N*,*N*,*O*-trimethylserine residue of the macrocycle are not important for the actin-depolymerizing activity.

**Scheme 11:**
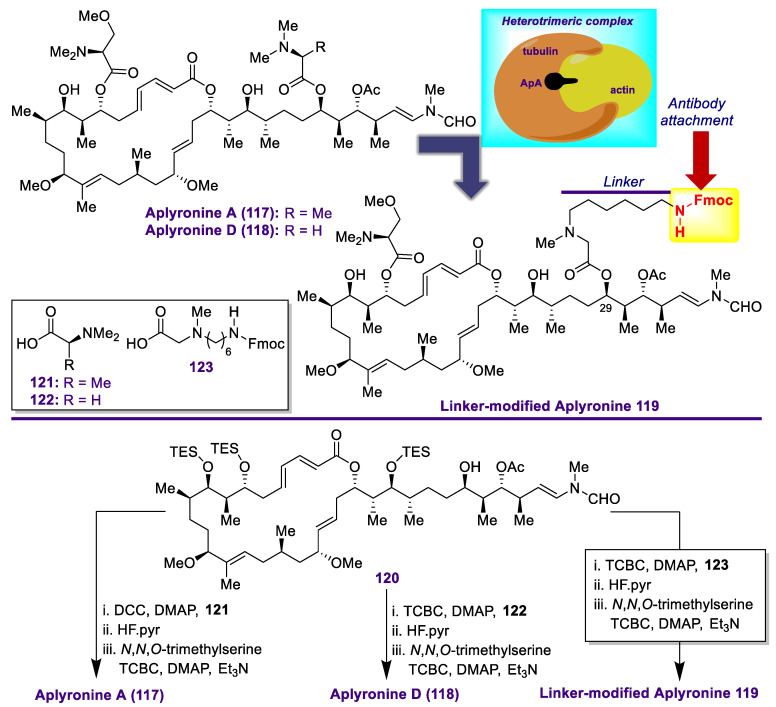
Total syntheses of aplyronines A (**117**) and D (**118**) and a potential payload **119** for ADCs.

Due to the interesting antitumoral activities of aplyronines A (**117**) and D (**118**), Paterson et al. designed a late-stage divergent synthetic strategy that allowed for their total synthesis from the common advanced precursor **120**, as depicted in Scheme 11 [178]. Moreover, from this intermediate, the linker-modified aplyronine **119** was efficiently synthesized, which was envisioned as a promising and valuable potential payload for the preparation of new ADCs based on these intriguing bioactive compounds. Thus, the functionalization was made on the secondary amine of the *N*-methylalanine residue of the side chain, introducing a 6-carbon atoms chain with an NHFmoc terminal for further assemblies. The incorporation of **119** as a payload in new ADCs is currently being investigated.

## 3. Conclusions

As amply demonstrated, ADCs are impressive therapeutics for cancer treatment that use antibodies as a vehicle to reach the tumor selectively, directing the drug to the desired cancer cells. This can be achieved via a smart design of ADCs, based on a linker that connects a cytotoxic payload with the antibody. This simple concept of ADC took decades to become a reality from the original Ehrlich’s idea of a ‘magic bullet’. Since then, intense research efforts have been devoted to the design, synthesis, and development of ADCs, and the combination of biology, biochemistry, and organic chemistry fields has been key to improving this technology over time, ultimately allowing for the introduction of ADCs into the clinic. Currently, there are more than 10 ADCs clinically approved and over 100 in clinical evaluation. In particular, a relevant type of ADCs is those containing a payload from a marine origin, which has been shown to possess unprecedented molecular structures together with impressive biological profiles. Through the present review, we have presented the molecular and biological diversity of ADCs containing payloads derived from marine sources, including in vitro and in vivo studies, as well as synthetic strategies for the assembly of the antibody, linker, and payload. In conclusion, the reader has hopefully realized a greater interest in this class of therapeutics and, in particular, the awesome power of the largely unexplored marine world, which highlights the potential of marine natural products as the vastest source of inspiration for the development of novel ADCs. The unlimited opportunities from the marine world, together with the great momentum of the ADC field, guarantee a golden future for this class of therapeutics. Hopefully, ADCs may revolutionize medicine in the near future by bringing new approved clinical drugs to treat cancer and other diseases with exceptionally high efficacy, delivery precision, and no side effects.

## Figures and Tables

**Table 1:** Payloads used in ADCs approved for market worldwide.

Payload	Origin	Mechanism of Action	ADC
Calicheamicin	Non-marine	DNA-damaging	Mylotarg, Besponsa
MMAE	Marine	Microtubule inhibitor	Adcetris, Polivy, Padcev, Tivdak
Maytansine DM1	Non-marine	Microtubule inhibitor	Kadcyla
*Pseudomonas* exotoxin 38	Non-marine	Elongation factor-2 inhibitor	Lumoxiti
Deruxtecan	Non-marine	DNA-damaging	Enhertu
Govitecan	Non-marine	DNA-damaging	Trodelvy
MMAF	Marine	Microtubule inhibitor	Blenrep
SG3199	Non-marine	DNA-damaging	Zynlonta

MMAE = Monomethyl auristatin E; MMAF = Monomethyl auristatin F.
